# Development of a Glycosaminoglycan Derived, Selectin Targeting Anti-Adhesive Coating to Treat Endothelial Cell Dysfunction

**DOI:** 10.3390/ph10020036

**Published:** 2017-03-29

**Authors:** James R. Wodicka, Andrea M. Chambers, Gurneet S. Sangha, Craig J. Goergen, Alyssa Panitch

**Affiliations:** 1 Weldon School of Biomedical Engineering, Purdue University, West Lafayette, IN 47907, USA; jwodicka@purdue.edu (J.R.W.); chambe48@purdue.edu (A.M.C.); gsangha@purdue.edu (G.S.S.); cgoergen@purdue.edu (C.J.G.); 2 Indiana University School of Medicine, Indianapolis, IN 46202, USA; 3 Department of Biomedical Engineering, University of California—Davis, Davis, CA 95616, USA

**Keywords:** glycocalyx, endothelial cell, dysfunction, selectin, dermatan sulfate, deep vein thrombosis

## Abstract

Endothelial cell (EC) dysfunction is associated with many disease states including deep vein thrombosis (DVT), chronic kidney disease, sepsis and diabetes. Loss of the glycocalyx, a thin glycosaminoglycan (GAG)-rich layer on the EC surface, is a key feature of endothelial dysfunction and increases exposure of EC adhesion molecules such as selectins, which are involved in platelet binding to ECs. Once bound, platelets cause thrombus formation and an increased inflammatory response. We have developed a GAG derived, selectin targeting anti-adhesive coating (termed EC-SEAL) consisting of a dermatan sulfate backbone and multiple selectin-binding peptides designed to bind to inflamed endothelium and prevent platelet binding to create a more quiescent endothelial state. Multiple EC-SEAL variants were evaluated and the lead variant was found to preferentially bind to selectin-expressing ECs and smooth muscle cells (SMCs) and inhibit platelet binding and activation in a dose-dependent manner. In an in vivo model of DVT, treatment with the lead variant resulted in reduced thrombus formation. These results indicate that EC-SEAL has promise as a potential therapeutic in the treatment of endothelial dysfunction.

## 1. Introduction

Deep vein thrombosis (DVT) affects approximately 900,000 individuals annually in the United States, and pulmonary emboli, a severe complication of DVT, are often observed in these patients [1]. Unlike in arterial thrombus, which is observed to initiate upon exposed extracellular matrix (ECM), venous thrombus initiates from dysfunctional endothelium, in part due to platelet binding to and activation on endothelium [1]. The binding and activation of platelets not only supports thrombus formation, but also further contributes to the inflammatory state of the venous endothelium, thus exacerbating the diseased vessel state [2].

Vascular endothelial cells (ECs) line the entire cardiovascular system in the human body. As the innermost layer of the blood vessel lumen, ECs are in constant contact with the blood and perform many critical functions. These include involvement in metabolism, regulating blood vessel tone, permeability and growth, vascular smooth muscle cell (SMC) proliferation, inflammation, platelet and leukocyte interactions, thrombosis and fibrinolysis [3,4,5]. Given these overlapping and wide-ranging activities, it is not surprising that disruption of normal EC function is associated with many different disease states. Indeed, EC dysfunction has been shown to play a role in DVT, atherosclerosis, myocardial infarction, peripheral vascular disease, stroke, hypertension, diabetes, chronic kidney disease, infections including sepsis, and even cancer [3,4,6,7,8,9,10,11].

In general, characteristics of endothelial dysfunction include a reduction in ability of vessels to vasodilate, along with increases in inflammation and prothrombotic properties [3,12]. Additionally, loss of the glycocalyx, an anionic layer comprised primarily of glycosaminoglycans (GAGs) that lines the endothelium, is a key feature of EC dysfunction [13,14]. The loss of this delicate layer causes a significant increase in vascular wall permeability and subsequent migration of fluid, protein and other cell types into the vessel [15]. Lack of a glycocalyx layer also results in exposure of EC adhesion molecules such as E-selectin, P-selectin, intracellular adhesion molecule 1 (ICAM-1), vascular cell adhesion molecule 1 (VCAM-1) and platelet-endothelial cell adhesion molecule 1 (PECAM-1) [13,16], some of which are known to play a role in platelet binding to the endothelial cell surface. Once bound, platelets release cytokines, chemokines and platelet activation factors, such as platelet activating factor (PAF), neutrophil-activating peptide-2 (NAP-2) and platelet factor-4 (PF-4), which leads to thrombus formation and a further increase in inflammatory cell recruitment to the site [17,18,19,20].

Endothelial dysfunction is likely a reversible disorder as eliminating cardiovascular risk factors through such means as smoking cessation, hypertension control, cholesterol lowering and physical activity have been shown to improve endothelial health [10]. Angiotensin converting enzyme (ACE) inhibitors, *N*-acetylcysteine (NAC), ascorbic acid (Vitamin C), vitamin E, and erythropoietin (EPO) have all been studied as potential therapeutics for EC dysfunction [21,22,23,24]. While some positive outcomes have been observed, they have often displayed a minimal effect on overall endothelial health or resulted in unintended cardiovascular events [4,23]. Therefore, additional means of targeting and effectively treating EC dysfunction and reducing associated cardiovascular events are needed.

Targeting EC adhesion molecules, such as selectins, for therapeutic or diagnostic purposes has been relatively well studied. Examples include microparticle drug delivery [25], therapeutic gene delivery [26,27], inflammation reduction [28,29,30], cancer metastasis prevention [31] and in vivo diagnostic imaging [32,33]. Specifically, antibodies and peptides have been utilized to target selectin receptors on EC surfaces to prevent platelet and leukocyte binding and the subsequent inflammatory response that occurs [28,29,30,34]. Therefore, our laboratory has recently developed several variants of a GAG derived, selectin targeting anti-adhesive coating (termed EC-SEAL) designed to utilize overexpressed selectins in EC dysfunction to prevent platelet binding and subsequent thrombus formation and restore a more quiescent endothelial state. Each variant consists of a dermatan sulfate (GAG component of the glycocalyx) [13] backbone with multiple selectin-binding peptides attached. EC-SEAL variants differing in type and number of peptides attached were investigated to determine highest binding affinities to inflamed ECs and assess ability to prevent platelet binding along the EC surface. Results indicated effective molecule binding to inflamed ECs and subsequent prevention of platelet binding and activation, as well as suppression of thrombus formation in a murine model of DVT, highlighting the possibility of EC-SEAL as a therapeutic to treat EC dysfunction.

## 2. Results

### 2.1. Selectin Expression

**Figure 1:**
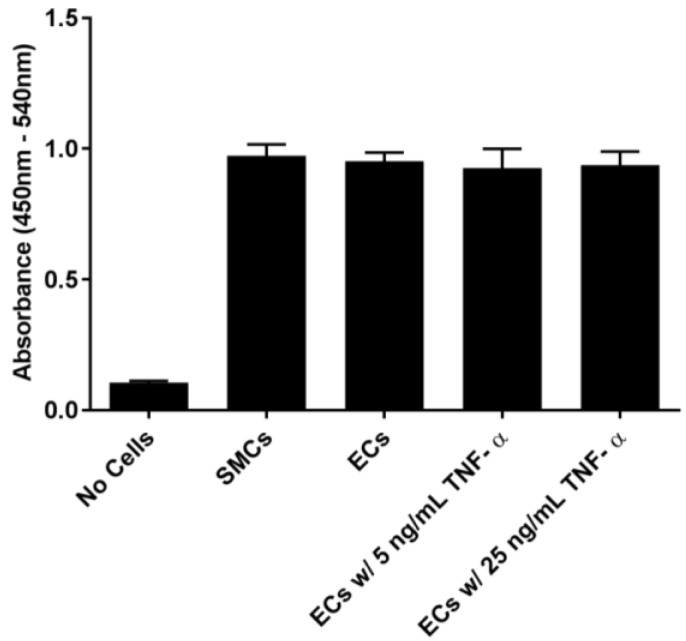
Selectin expression on SMCs and ECs under various conditions. Expression was quantified using primary anti-E-selectin and secondary horseradish peroxidase (HRP)-conjugated antibodies. All cultures exhibited significant selectin expression; however, selectin levels did not change when stimulated with TNF-α. *n* = 3; *p* < 0.05.

To measure levels of selectin expression on SMCs and ECs under various conditions, antibodies designed to target E-selectin were utilized. ECs were stimulated with 5 or 25 ng/mL tumor necrosis factor (TNF)-α or control (unstimulated) media, and SMCs were stimulated with only control (unstimulated) media. While all cultures showed selectin expression compared to the background (No Cells), there was no difference in expression between cultures that contained TNF-α stimulated and control media (Figure 1). Since antibodies are likely cross-reactive with P-selectin, these data suggest SMCs, which have been shown to express P-selectin [35,36] but not E-selectin [37], also exhibited selectin expression. Both lower and higher concentrations of TNF-α (Supplementary Figure S1) and other proinflammatory cytokines (interleukin (IL)-1β, IL-6, lipopolysaccharide (LPS)) at varying concentrations (10–100 ng/mL) (Supplementary Figure S2) were tested in an attempt to upregulate selectin expression compared to unstimulated ECs. All proinflammatory stimuli failed to show any upregulation in selectin expression, indicating that EC cultures likely exhibited a basal upregulation of selectin receptors. Additionally, ECs were unable to form fully intact monolayers despite a lack of proinflammatory stimuli present, as indicated by their permeability to fluorescently-labeled dextran (Supplementary Figure S3). This lack of cell–cell junctions under normal conditions is another indication that the cultured ECs were likely in a constitutively activated state.

### 2.2. EC-SEAL Binding (Cells)

**Figure 2:**
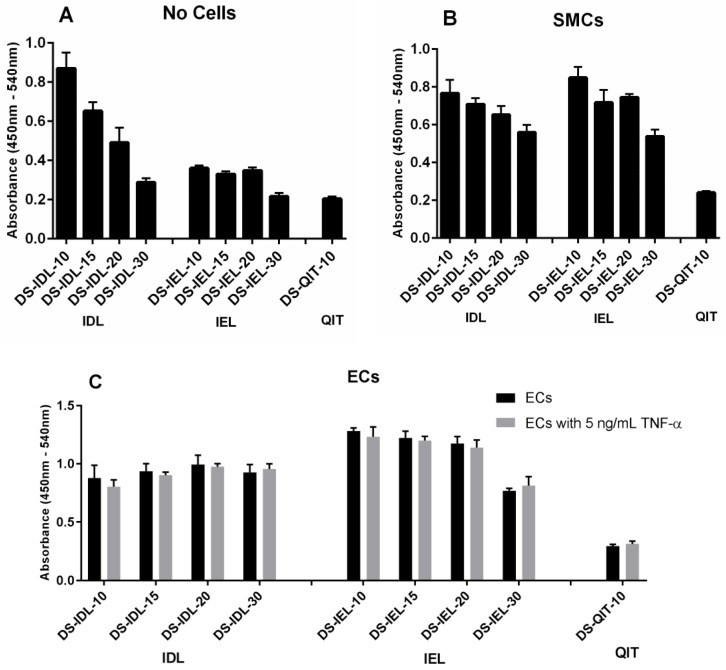
EC-SEAL variant binding to No Cells, SMCs and ECs (with and without 5 ng/mL TNF-α). (**A**) No Cells; (**B**) SMCs; and (**C**) ECs with and without 5 ng/mL TNF-α were treated with 3 μM of each EC-SEAL variant. Absorbance was measured using a streptavidin-HRP assay to quantify biotinylated peptides on each molecule bound to the surface. None of the EC-SEAL variants displayed a significant difference in binding to unstimulated ECs and ECs stimulated with TNF-α. Variants containing the peptide IEL showed the highest binding affinity to ECs. *n* = 3; *p* < 0.05.

Each selectin-binding peptide being examined (IDLMQARGC (IDL), IELLQARGC (IEL) and QITWAQLWNMMKGC (QIT)) was used to synthesize EC-SEAL variants containing 10, 15, 20 and 30 peptides per dermatan sulfate (DS) backbone (with, on average, one biotinylated peptide per DS molecule). To test the binding ability of these EC-SEAL molecules varying in both type and number of peptides, binding to SMCs and ECs (with and without 5 ng/mL TNF-α) was examined. Results of our first analysis are shown in Figure 2. Overall, both the peptide itself and the number of peptides attached to each molecule affected binding affinity. We observed that none of the variants tested displayed a difference in binding when added to ECs in control (unstimulated) media versus ECs stimulated with 5 ng/mL TNF-α (Figure 2C). This was consistent with data presented in Figure 1 in which ECs exhibited the same level of selectin expression with or without TNF-α stimulation. In addition, all variants (except DS-IDL_10_) exhibited a higher affinity for ECs than SMCs (Figure 2B,C). EC-SEAL variants containing the IDL peptide appeared to have increased nonspecific binding, particularly in molecules with a low number of peptides, as indicated by the relatively high binding in the absence of cells (Figure 2A). Additionally, variants containing the peptide IEL displayed the highest level of binding to ECs with and without TNF-α stimulation (Figure 2C). It should be noted that QIT variants with 15, 20 and 30 peptides per DS were also tested in other studies and showed similar binding as DS-QIT_10_, but due to solubility issues leading to difficulty in molecule synthesis, use of these variants was discontinued.

**Figure 3:**
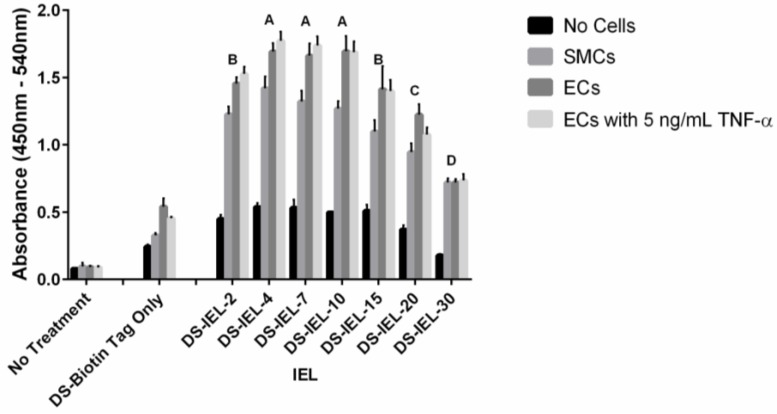
IEL variant binding to No Cells, SMCs and ECs (with and without 5 ng/mL TNF-α). Concentration of IEL variants = 3 μM. Absorbance was measured using a streptavidin-HRP assay to quantify biotinylated peptides on each molecule remaining bound to the surface. Each letter represents groups that exhibited statistically equivalent binding to ECs. Number of molecules bound to EC surface: A > B > C > D. *n* = 2; *p* < 0.05.

Given its superior ability to bind to ECs, further investigations focused on EC-SEAL variants containing the peptide IEL. Since DS-IEL_10_ displayed some of the highest levels of binding during initial testing, variants with even fewer peptides were synthesized and evaluated. Figure 3 depicts a comparison of two controls (No Treatment and DS-Biotin Tag Only) and all synthesized IEL variants ranging from 2 to 30 peptides per DS backbone. There was again no difference between binding to unstimulated ECs versus ECs stimulated with 5 ng/mL TNF-α, regardless of treatment applied. All variants demonstrated an increased binding affinity to ECs over SMCs (with the exception of DS-IEL_30_) and all IEL variants showed increased binding compared to the DS-Biotin Tag Only control. As indicated in Figure 3, DS-IEL_4_, DS-IEL_7_ and DS-IEL_10_ had the highest number of molecules bound to ECs and were statistically equivalent to each other. Therefore, DS-IEL_10_ was chosen as the molecule for further testing given that it contained the most selectin-binding peptides among the highest binding group.

### 2.3. Platelet Activation and Binding

**Figure 4:**
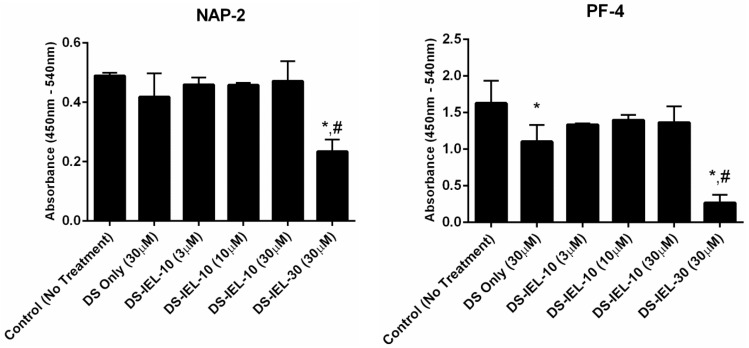
Impact of DS-IEL_10_ and DS-IEL_30_ on platelet activation markers NAP-2 and PF-4. ECs were stimulated with 5 ng/mL TNF-α and treated with 3–30 µM EC-SEAL variants or control (No Treatment). PRP was added and NAP-2 and PF-4 levels in the collected PRP were recorded using sandwich ELISAs. DS-IEL_30_ showed the greatest reduction in platelet activation. * represents a significant difference from the control (No Treatment); ^#^ represents a significant difference when compared to all other treatment groups. *n* = 3; *p* < 0.05.

After confirming EC-SEAL variants not only bound to cell surfaces, but also their binding was dependent on type and number of peptides attached, their ability to prevent platelet binding and subsequent activation was quantified. We first investigated DS-IEL_10_ because, based on its increased level of binding to endothelial cells (Figure 3), we expected that it would best inhibit platelet binding as compared to the other variants we examined. DS-IEL_30_ was included to verify that the IEL peptide would not enhance platelet binding to the activated endothelium due to its interaction with the P-selectin receptor that is present on platelets. Levels of NAP-2 and PF-4, released from platelets during activation [18,20,38], were used as platelet activation markers to compare various treatment conditions. Surprisingly, treating ECs with DS-IEL_10_ prior to platelet rich plasma (PRP) incubation had no effect on NAP-2 and PF-4 release as compared to the control (No Treatment), regardless of concentration of DS-IEL_10_ applied (Figure 4). However, treating with 30 µM DS-IEL_30_ showed a significant decrease in NAP-2 and PF-4 levels when compared to the control and all other treatment groups.

**Figure 5:**
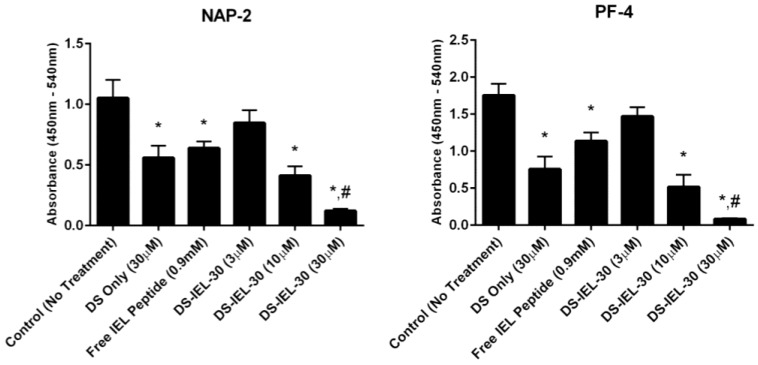
Dose-dependent response of DS-IEL_30_ on platelet activation. ECs were stimulated with 5 ng/mL TNF-α and treated with 3–30 µM DS-IEL_30_ or control (No Treatment). PRP was added and NAP-2 and PF-4 levels in the collected PRP were recorded using sandwich ELISAs. DS-IEL_30_ at 30 µM proved to be the most effective dose to reduce platelet activation. * represents a significant difference from the control (No Treatment); # represents a significant difference when compared to all other treatment groups. *n* = 2; *p* < 0.05.

Next, various concentrations of DS-IEL_30_ were tested to determine an effective dose. As shown in Figure 5, DS-IEL_30_ decreases platelet activation markers at 10 µM compared to the control (No Treatment), however, treating with 30 µM DS-IEL_30_ results in a greater decrease in both NAP-2 and PF-4 compared to the control and all other treatment groups. The individual components of DS-IEL_30_, DS only (30 µM) and free IEL peptide (0.9 mM), were also tested at concentrations equivalent to 30 µM DS-IEL_30_. While both components decreased platelet activation markers compared to the control, neither did so as effectively as 30 µM DS-IEL_30_.

**Figure 6:**
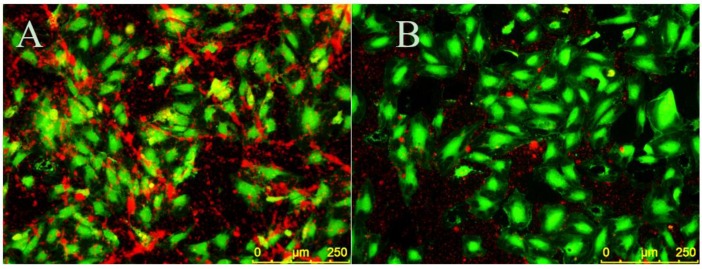
Platelets binding to ECs in the presence of EC-SEAL. ECs (green) were stimulated with 5 ng/mL TNF-α and treated with: control media (**A**); or 30 µM DS-IEL_30_ (**B**). Platelets (red) were isolated from human whole blood and incubated on ECs. Cultures treated with DS-IEL_30_ showed less platelet binding, particularly on EC surfaces. The 10× images were acquired using a fluorescent microscope. Scale bar = 250 µm. *n* = 3.

In addition to reducing platelet activation, DS-IEL_30_ was also shown to prevent platelet binding to ECs (Figure 6). Labeled with membrane bound CellTrackers, platelets (red) were added to ECs (green) with and without prior 30 µM DS-IEL_30_ treatment. Treated EC cultures (Figure 6B) displayed a decrease in overall platelet binding, particularly directly on ECs, compared to untreated cultures (Figure 6A). In addition, the limited number of platelets that did bind to cultures treated with DS-IEL_30_ appeared to bind in areas not covered by ECs, indicating that DS-IEL_30_ was preferentially targeting ECs (and not the underlying tissue culture plastic) and subsequently preventing platelet binding and activation.

### 2.4. EC-SEAL Binding (Selectin Protein)

**Figure 7:**
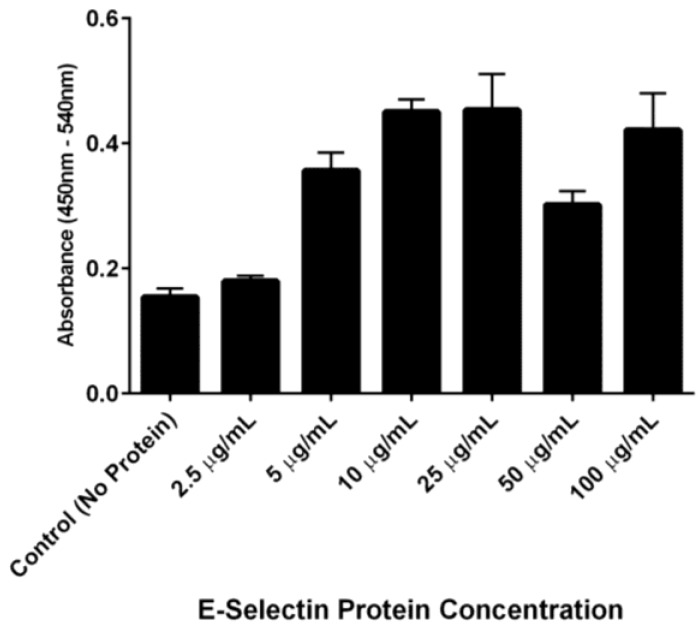
EC-SEAL binding to recombinant human E-selectin. Absorbance was measured using a streptavidin-HRP assay to quantify biotinylated peptides on DS-IEL_30_ bound to the surface. DS-IEL_30_ exhibits preferential binding to selectin over BSA and is dependent upon the concentration of selectin protein present. *n* = 3; *p* < 0.05.

To confirm that the observed interactions between EC-SEAL variants and cells were through a selectin binding process, the ability of DS-IEL_30_ to preferentially bind to selectin protein was studied. Increasing concentrations of human recombinant E-selectin were coated on a high bind plate and 1% bovine serum albumin (BSA) was added to eliminate nonspecific binding. A single concentration of DS-IEL_30_ (3 µM) was incubated and the amount bound to the surface was quantified (Figure 7). Initially, DS-IEL_30_ binding increases with increased concentrations of E-selectin. Then, at 50 µg/mL E-selectin, DS-IEL_30_ binding falls off slightly, but is overcome with further increases in E-selectin concentration (100 µg/mL).

**Figure 8:**
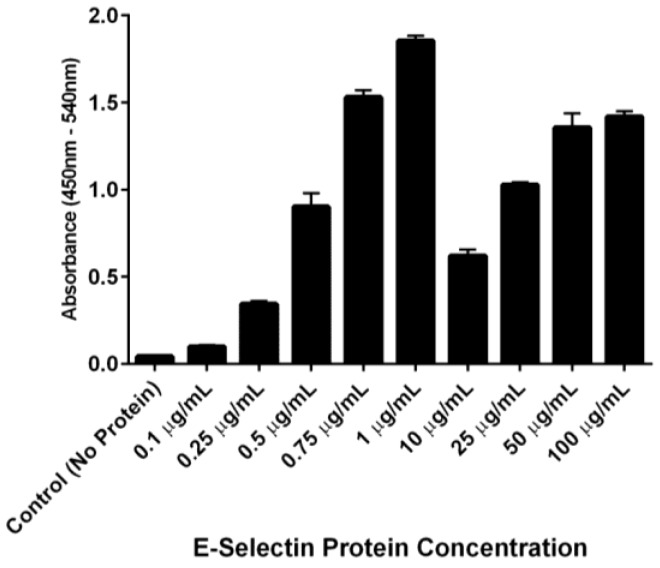
Antibody binding to recombinant human E-selectin. Expression was quantified using primary anti-E-selectin and secondary HRP-conjugated antibodies. Antibodies display preferential binding to selectin over BSA and binding is dependent upon the concentration of selectin protein present. *n* = 2; *p* < 0.05.

A similar trend is observed when the binding of antibodies (primary mouse monoclonal anti-E-selectin IgG_2a_ with secondary goat anti-mouse IgG HRP-conjugated) are quantified under the same conditions (Figure 8). There is also an initial increase in antibody binding with increased concentrations of E-selectin. Then, at 10 µg/mL E-selectin, antibody binding falls off significantly, but is also overcome with increasing concentrations of E-selectin (25–100 µg/mL).

### 2.5. DVT Mouse Model

**Figure 9:**
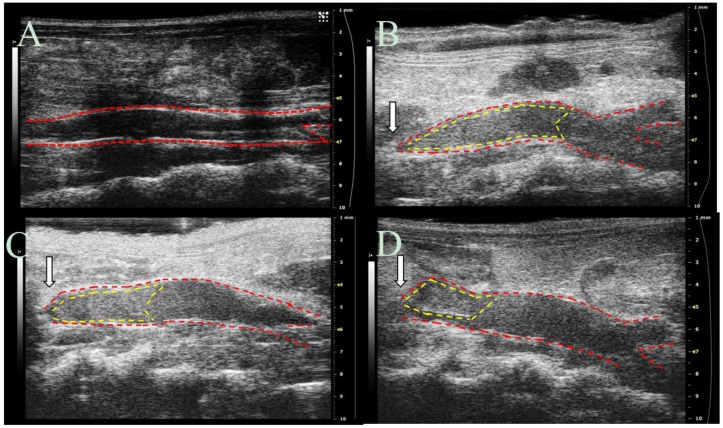
B-Mode ultrasound images of the IVC before and after partial ligation surgery. Representative images from: (**A**) Baseline (pre-surgery); (**B**) Saline; (**C**) Heparin; and (**D**) EC-SEAL (DS-IEL_30_) at 6 h after partial ligation are shown. IVC is outlined in red and thrombus is outlined in yellow. White arrows represent the location of the 6-0 silk suture creating the partial ligation.

**Figure 10:**
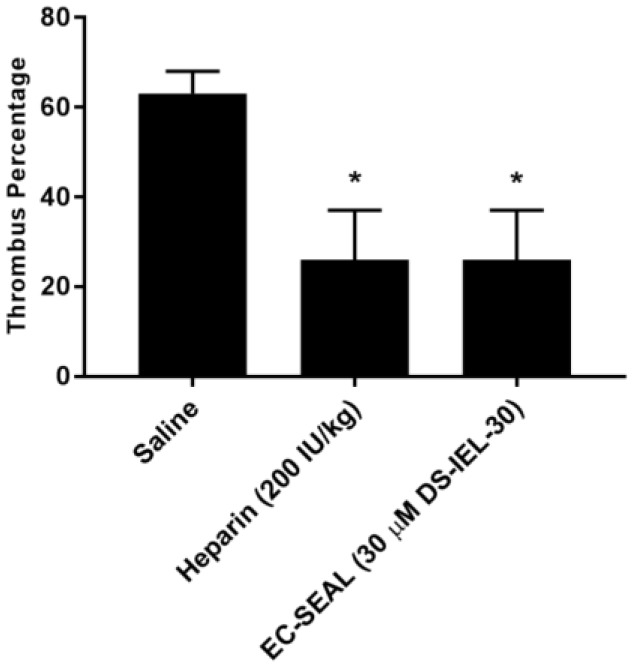
Thrombus percentage in DVT mouse model. Ultrasound images were utilized to calculate thrombus percentage by dividing thrombus volume by total IVC volume between the partial ligation suture and iliac bifurcation. Both heparin (*n* = 6) and EC-SEAL (*n* = 4) decreased thrombus percentage compared to saline controls (*n* = 6). * represents a significant difference from the control group (Saline). *p* < 0.05.

To assess the effects of DS-IEL_30_ in vivo, a study utilizing a mouse model of DVT was conducted. Partial ligation of the inferior vena cava (IVC) was performed and tail vein injections of saline (*n* = 6), 200 IU/kg heparin (*n* = 6), or 30 µM DS-IEL_30_ (*n* = 4) were administered. Ultrasound images were obtained at 6 h post-ligation for each mouse (Figure 9). The thrombus volume (expressed as mean ± standard error of the mean) was 12.3 ± 1.6 mm^3^ for saline, 5.0 ± 2.2 mm^3^ for heparin, and 4.3 ± 2.2 mm^3^ for DS-IEL_30_. The mean thrombus percentage (defined as thrombus volume/total IVC volume between the ligation suture and iliac bifurcation) was 63% ± 5% for saline, 26% ± 11% for heparin, and 26% ± 11% for DS-IEL_30_ (Figure 10). Both heparin and DS-IEL_30_ significantly decreased thrombus percentage compared to the saline controls (*p* < 0.05).

### 2.6. Clotting Time (aPTT)

**Figure 11:**
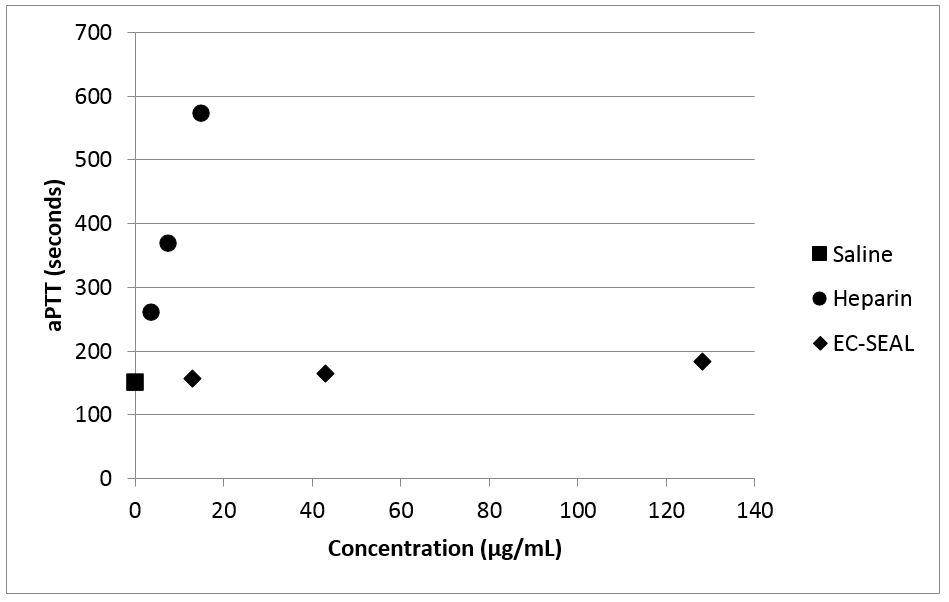
Whole blood clotting time (aPTT) in the presence of heparin and EC-SEAL. aPTT measurements were recorded with varying concentrations of heparin and EC-SEAL. Heparin exhibited a much greater effect on aPTT than EC-SEAL, likely indicating different mechanisms of reducing thrombus formation in vivo. *n* = 2; *p* < 0.05.

Although heparin and EC-SEAL (DS-IEL_30_) showed statistically equivalent outcomes with respect to thrombus percentage in the DVT mouse model, it is believed these effects are achieved through different mechanisms. Therefore, clotting times (activated partial thromboplastin time (aPTT)) were obtained for various concentrations of heparin and EC-SEAL (Figure 11). It should be noted that the highest concentration of both heparin and EC-SEAL depicted in Figure 11 were equivalent to the doses administered to mice in vivo. Heparin displayed a substantial effect, causing a nearly four-fold increase in aPTT at the highest dose compared to saline controls. Comparatively, EC-SEAL exhibited a relatively small effect on aPTT (e.g., 23% increase at the highest concentration compared to saline controls).

## 3. Discussion

Loss of the glycocalyx is a key characteristic of EC dysfunction [13,14]. Removal of this glycosaminoglycan (GAG)-rich layer results in the exposure and upregulation of EC adhesion molecules, including selectins [13,16]. Selectins and their ligands, located on both ECs and platelets, play a key role in the binding of platelets to inflamed endothelium. In the context of endothelial dysfunction, this process can lead to complications in a myriad of disease states including DVT, atherosclerosis, diabetes, chronic kidney disease and sepsis [3,4,6,8,39]. Masking these selectins with antibodies or peptides to prevent platelet binding presents a possible avenue of treatment [28,29,30]. We sought to develop and test multiple variants of a GAG derived, selectin-binding anti-adhesive coating (termed EC-SEAL) utilizing selectin-binding peptides to bind to an inflamed endothelial surface. We demonstrate the ability to successfully synthesize variants ranging from 2 to 30 peptides per dermatan sulfate molecule that bind to E-selectin and cells expressing selectins, and prevent platelet binding and activation in a dose-dependent manner. Additionally, we present evidence that the lead variant reduces thrombus size in vivo while exhibiting minimal effects on clotting time.

In order to examine the binding of EC-SEAL variants to vascular cells, we first established that selectin was present on the cell surfaces. As shown in Figure 1 and Supplementary Figures S1 and S2, ECs (which express both E and P-selectin) did not increase selectin levels following TNF-α stimulation or any other proinflammatory stimulants used. Although not anticipated because it has been shown previously that adding TNF-α to human aortic ECs leads to an increase in E-selectin expression 3–6 h after stimulation [40,41,42], there is also evidence that many different factors including number of cell passages, seeding density, certain growth factors present in media, and even the culture substrate can lead to increased basal levels of adhesion molecules on cultured ECs [43,44,45,46]. It is also possible that the rinsing and manipulation of the live ECs throughout the experiments contributed to the observed basal selectin expression. The presence of selectins on the EC surface in the unstimulated cultures, the lack of response to multiple proinflammatory cytokines (TNF-α, LPS, IL-1β and IL-6), and the inability to form intact monolayers (Supplementary Figure S3), all suggest that the EC cultures were in a constitutively activated state. SMCs, known to express P-selectin [35,36], but not E-selectin [37], demonstrated P-selectin cross-reactivity with the anti-E-selectin antibody.

The selectin-expressing cells were then used to test and compare EC-SEAL variant binding. Both the type and number of peptides attached to the DS backbone had a significant impact on binding affinity (Figure 2 and Figure 3). There was no difference in binding to unstimulated ECs or ECs stimulated with TNF-α for any EC-SEAL variant, which is consistent with the selectin expression data discussed previously. Although nearly all variants had a higher binding affinity to ECs, they also adhered to SMCs, presumably through P-selectin. Prior studies on IDL and IEL peptides indeed indicated binding to both E and P-selectin [31]. QIT was previously shown to have a significantly higher preference for E-selectin over P-selectin [47], but in our hands the binding observed in the DS-QIT_x_ variants was primarily due to nonspecific interactions as indicated by the (relatively) high level of binding with no cells present compared to both SMCs and ECs (Figure 2). IEL was selected as the peptide to examine further due to its increased binding to ECs and reduced nonspecific interactions when no cells were present. This is consistent with a previous study that showed IEL had a higher binding affinity to E-selectin expressing bacteria than IDL [31]. Importantly, all DS-IEL_x_ variants showed increased levels of binding compared to only DS, which as a native glycocalyx component will interact with the EC surface, indicating the benefit of the selectin-binding peptides. The apparent ability to bind to both E and P-selectin is also viewed as advantageous as it may allow for more complete coverage of: (1) ECs expressing both E and P-selectin; and (2) SMCs expressing P-selectin should they become exposed following EC injury or denudation.

DS-IEL_10_ was originally chosen for platelet binding and activation testing given that it possessed the greatest number of selectin-binding peptides among the highest binding group of DS-IEL_x_ variants (Figure 3.) However, using NAP-2 and PF-4 as platelet activation markers, DS-IEL_10_ did not reduce platelet activation at any treatment concentration compared to untreated controls (Figure 4). DS-IEL_30_ was also studied because the high density of selectin-binding peptide per backbone presented the possibility that some peptide would not be bound to the endothelial cell surface and might in fact bind to P-selectin on platelets and increase platelet binding. Importantly, there was no evidence for platelet-peptide interactions in these studies; in fact, DS-IEL_30_ showed a dose-dependent response in reducing platelet activation compared to untreated controls (Figure 5). Additionally, 30 µM DS-IEL_30_ proved to be an extremely effective dose even when compared to equivalent amounts of its individual components (30 µM DS Only and 0.9 mM Free IEL Peptide). We believe that the apparent discrepancy between the binding studies (where DS-IEL_10_ was believed to be best) and platelet activation results (where DS-IEL_30_ was most effective) lies in how the binding was quantified. Since each DS backbone has one biotinylated peptide, the streptavidin-HRP assay will quantify each DS molecule attached regardless of the number of selectin-binding peptides bound to the surface. Therefore, despite DS-IEL_10_ showing a slightly more than two-fold increase in number of molecules bound compared to DS-IEL_30_, there are three times the number of selectin-binding peptides on each molecule of DS-IEL_30_, resulting in a higher number of peptides available to bind to the cell surface and block interaction with platelets. Furthermore, increasing the number of peptides per molecule appeared to decrease nonspecific interactions (Figure 2 and Figure 3) and therefore, more of the molecules likely reached and bound to their targeted site. This suggests that despite fewer molecules present, the higher local peptide density of DS-IEL_30_ supports more efficient binding to the EC surface to prevent platelet binding and activation.

The ability of DS-IEL_30_ to preferentially bind to recombinant human E-selectin over BSA was observed by incubating a single concentration of DS-IEL_30_ on plates coated with increasing concentrations of E-selectin. DS-IEL_30_ binding increased with initial increases in E-selectin concentration, but dropped off at a certain point, only to increase again upon a further increase in E-selectin concentration (Figure 7). Interestingly, anti-E-selectin antibodies exhibited a similar binding trend, although at different concentrations of E-selectin (Figure 8). We hypothesize that these binding characteristics are due to a potential selectin conformational change that is dependent upon concentration of both receptor (E-selectin) and ligand (DS-IEL_30_ or antibody) and their respective binding affinities. Ligand binding itself is known to cause conformational changes at the binding site [48,49], including that of E-selectin [50,51]. It is also possible that the concentration of E-selectin protein coated on the plate’s surface influences the orientation in which the protein adsorbs and therefore, the exposure (or lack thereof) of ligand binding sites. Given that DS-IEL_30_ and anti-E-selectin antibody may have different binding sites and affinities to E-selectin, it is not surprising that the drop off and subsequent rise in binding occur at different concentrations of plated E-selectin protein.

Given the vast number of vascular disease states associated with endothelial dysfunction, animal models in this area are wide-ranging. The utilization of mouse models has proven to be an extremely useful tool allowing for the study of underlying mechanisms and initial screenings of therapeutics [52]. Thus, we employed a mouse model of deep vein thrombosis mediated endothelial dysfunction to observe the anti-thrombotic effects of EC-SEAL in vivo. This inferior vena cava (IVC) partial ligation model provides valuable information regarding vessel wall-blood interactions during thrombus formation and has been used previously to evaluate therapeutic agents [52,53]. Study results, obtained via ultrasound imaging, indicate that DS-IEL_30_ significantly decreases thrombus formation at six hours following IVC partial ligation (Figure 10) and highlight the potential for DS-IEL_30_ to act locally following systemic administration. It is believed that these therapeutic effects are due to DS-IEL_30_ binding to the vessel wall and subsequently preventing platelet binding and not through direct anticoagulant properties. Dermatan sulfate from porcine origin (which is the type used for EC-SEAL synthesis) has been shown to have very minimal anticoagulant properties depending on the concentration [54]. By measuring aPTT of whole blood that had been incubated with varying concentrations of both heparin and DS-IEL_30_, we have confirmed the minimal effect of DS-IEL_30_ on clotting time (Figure 11). Therefore, EC-SEAL has the potential to be utilized as a treatment for DVT without the negative side effects of current standard of care anticoagulant therapies such as heparin.

Since DS-IEL_30_ proved to be the most effective EC-SEAL variant and no variants were synthesized with greater than 30 peptides per DS molecule, it is possible that further increases to the number of peptides per molecule may be beneficial. Previous work has shown, however, that excessive oxidation of GAGs can lead to chain scission [55,56], so a balance between number of selectin-binding peptides and DS backbone integrity will need to be considered. It is also possible that targeting additional EC adhesion molecules, such as ICAM-1 or VCAM-1, may further increase binding and therapeutic effect. Future studies on DS-IEL_30_ and any additional EC-SEAL variants can focus on prevention of leukocyte binding and subsequent diapedesis. Additional experiments further assessing the specificity of EC-SEAL binding as well as observing the binding characteristics of EC-SEAL over time and under flow conditions to account for shear forces would be valuable.

## 4. Materials and Methods

### 4.1. GAG Dervied, Selectin Targeting Anti-Adhesive Coating (EC-SEAL) Synthesis

**Scheme 1:**
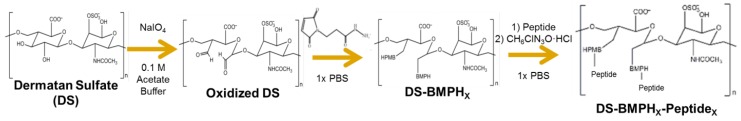
GAG-derived, selectin targeting anti-adhesive coating (EC-SEAL) synthesis.

The synthesis of the GAG derived, selectin targeting anti-adhesive coating variants (Scheme 1) was performed at room temperature as follows: vicinal diol groups on dermatan sulfate (DS, MW_avg_ 46,275 Da, Celsus Laboratories, Cincinnati, OH, USA) were oxidized to aldehydes using sodium meta-periodate (Thermo Fisher Scientific, Waltham, MA, USA) in 0.1 M acetate buffer. The ratio of DS to sodium meta-periodate varied depending on the desired degree of oxidation. Following oxidation, the modified DS was isolated via size exclusion chromatography (SEC) (AKTA Purifier FPLC, GE Healthcare). An excess of *N*-[β-maleimidopropionic acid] hydrazide, trifluoroacetic acid salt (BMPH, Thermo Fisher Scientific), was then conjugated via the hydrazide to the DS aldehyde groups in 1× phosphate buffered saline (PBS, 1.05 mM KH_2_PO_4_, 155.17 mM NaCl, 2.97 Na_2_HPO_4_-7H_2_O; pH 7.4, Thermo Fisher Scientific) to form DS-BMPH_x_. Unbound BMPH was removed via SEC and quantified by calculating the area under the peak to determine the average number (*x*) of BMPH molecules bound to each DS backbone. Next, biotinylated peptide was added at a 1:1 molar ratio of DS:biotinylated peptide in 1× PBS to conjugate an average of one biotinylated peptide per molecule of DS, and then an excess of one of three selectin-binding peptides (Genscript, Piscataway, NJ, USA) was conjugated via the cysteine thiol to the maleimide groups on DS-BMPH_x_ in 1× PBS. The three peptide sequences used were as follows: IDLMQARGC (IDL) [31], IELLQARGC (IEL) [31,32,34,57], and QITWAQLWNMMKGC (QIT) [47]. After the peptide reaction was complete, semicarbazide hydrochloride (Sigma-Aldrich, St. Louis, MO, USA) was added directly to the solution to reduce any unreacted aldehyde groups. Subsequent SEC isolation and lyophilization resulted in the purified sixteen variants containing from one to thirty peptides per DS backbone of DS-BMPH_x_-Peptide_x, biotin_ (hereby simply referred to in the form of DS-Peptide_x_, ex: DS-IEL_20_).

### 4.2. Cell Cultures

Human aortic endothelial cells (ECs, Thermo Fisher Scientific) were cultured in Medium 200 (M 200, Thermo Fisher Scientific) with low serum growth supplement (LSGS, Thermo Fisher Scientific). Human coronary artery smooth muscle cells (SMCs, Thermo Fisher Scientific) were cultured in Medium 231 (M 231, Thermo Fisher Scientific) with smooth muscle growth supplement (SMGS, Thermo Fisher Scientific). Cells were maintained at 37 °C and 5% CO_2_. Unless otherwise noted, passage 3–7 cells were seeded at a density of 1 × 10^5^ cells/cm^2^ on tissue culture polystyrene and allowed to adhere for 24 h prior to any stimulation or treatment.

### 4.3. Selectin Expression

Monolayers of SMCs and ECs were seeded separately on Biocoat Collagen I Cellware 96-well plates (Corning, Corning, NY, USA). ECs were stimulated for four hours with 0.05–50 ng/mL tumor necrosis factor-α (TNF-α, Thermo Fisher Scientific), 10–100 ng/mL interleukin-1β (IL-1β, PeproTech, Rocky Hill, NJ, USA), 10–100 ng/mL interleukin-6 (IL-6, PeproTech), 10–100 ng/mL lipopolysaccharide (LPS, Sigma-Aldrich) or control (unstimulated) media. SMCs were cultured with only control (unstimulated) media. To block nonspecific binding, cells were treated with 1% bovine serum albumin (BSA, Sigma-Aldrich) in 0.05 M tris-buffered saline (TBS, 0.138 M NaCl, 0.0027 M KCl; pH 8.0, Sigma-Aldrich) for 30 min at room temperature with shaking. Primary rabbit polyclonal anti-E-selectin IgG (Santa Cruz Biotechnology, Dallas, TX, USA) or mouse monoclonal anti-E-selectin IgG_2a_ (Santa Cruz Biotechnology) antibody was added (1:50 dilution of initial 200 μg/mL in 1% BSA in TBS) for one hour at 4 °C with shaking. Cells were rinsed three times with 1% BSA in TBS and secondary donkey anti-rabbit IgG HRP-conjugated (Thermo Fisher Scientific) or goat anti-mouse IgG HRP-conjugated (Thermo Fisher Scientific) antibody was added (1:1000 dilution of initial 0.5 mg/mL in 1% BSA in TBS) for 30 min at room temperature with shaking. Cells were rinsed three times with 1% BSA in TBS and colorimetric change was induced with 1:1 hydrogen peroxide: tetramethylbenzidine (R&D Systems, Minneapolis, MN, USA). Following 20 min of incubation with shaking, 2N sulfuric acid was added to cease the reaction and absorbance was measured at 450 nm and 540 nm using a SpectraMax M5 plate reader (Molecular Devices, Sunnyvale, CA, USA). For each individual well, 540 nm was subtracted from 450 nm to obtain final absorbance reading.

### 4.4. EC Monolayer Permeability

ECs were seeded in control (unstimulated) media on tissue culture treated Transwell polyester membrane inserts with 3.0 μm pores on a 24-well plate (Corning) and cultured for 48 h to form monolayers. Cell culture media in both the upper and lower chambers was then changed with fresh, unstimulated media, and Rhodamine B isothiocyanate (RITC)-dextran (Sigma-Aldrich) was added to obtain a final concentration of 3 mg/mL in the upper chamber. The monolayers were incubated at 37 °C and 5% CO_2_ for four hours, then the media in the lower chamber was removed and fluorescence was read using an M5 plate reader (Ex: 520 nm; Em: 590 nm).

### 4.5. EC-SEAL Binding to Cells

Monolayers of SMCs and ECs were seeded separately on Biocoat Collagen I Cellware 96-well plates or CellBIND surface 96-well plates (Corning). ECs were stimulated for four hours with 5 ng/mL TNF-α or control (unstimulated) media, and SMCs were treated with only control (unstimulated) media. Nonspecific binding was blocked using 1% BSA in TBS with 150 mM CaCl_2_ for 20 min at room temperature with shaking. Cells were then treated with EC-SEAL variants (3 µM in 1% BSA in TBS with 150 mM CaCl_2_) for one hour at 37 °C and 5% CO_2_. After rinsing three times with 1% BSA in TBS with 150 mM CaCl_2_, streptavidin-HRP (R&D Systems) was diluted 1:200 in 1% BSA in TBS with 150 mM CaCl_2_ and added for 20 min at room temperature with shaking. After three more rinses in TBS with 150 mM CaCl_2_, a 1:1 hydrogen peroxide: tetramethylbenzidine solution was added to induce colorimetric change. After 20 min of incubation with shaking, 2N sulfuric acid was used to stop the reaction and an M5 plate reader was utilized to measure absorbance at 450 nm and 540 nm as described above.

### 4.6. Platelet Activation

EC monolayers were seeded on Biocoat Collagen I Cellware 96-well plates and stimulated with 5 ng/mL TNF-α for four hours. One-percent BSA in TBS with 150 mM CaCl_2_ was added for 15 min at room temperature to decrease nonspecific binding. ECs were then treated with 3–30 µM EC-SEAL variants for 1 h at 37 °C and 5% CO_2_. Whole blood was obtained from healthy volunteers in collection vials containing sodium citrate and centrifuged for 20 min at 200× *g* and 25 °C. Platelet rich plasma (PRP) was isolated and after EC-SEAL treatments, ECs were rinsed three times with TBS with 150 mM CaCl_2_ and 100 µL of PRP was added to each well. After 1 h of incubation at room temperature, 45 µL of PRP was then removed and added to tubes containing 5 µL of ETP (107 mM Ethylenediaminetetraacetic acid, disodium salt (EDTA, Promega, Madison, WI, USA), 12 mM Theophylline (Sigma-Aldrich) and 2.8 mM Prostaglandin E1 (Enzo Life Sciences, Farmingdale, NY, USA) in water). Samples were then centrifuged for 30 min at 2000× *g* and 4 °C and supernatant was collected and stored at −80 °C for use in subsequent assays.

### 4.7. NAP-2 and PF-4 ELISA

To quantify platelet activation, neutrophil activating peptide-2 (NAP-2) and platelet factor-4 (PF-4) levels in PRP collected from platelet activation experiments were measured utilizing sandwich ELISAs. Mouse monoclonal anti-hNAP-2 IgG and anti-hPF-4 IgG_2B_ capture antibodies (R&D Systems) were coated on 96-well EIA/RIA high binding plates (Corning) at 2 µg/mL in 1× phosphate buffered saline (PBS) and incubated at 4 °C overnight. After rinsing three times with 1× PBS + 0.05% Tween and blocking with 1% BSA in 1× PBS for 1 h at room temperature with shaking, previously collected PRP samples were thawed, diluted 1:10,000 in 1% BSA in 1× PBS and added for 2 h at room temperature with shaking. Samples were removed, wells were triple rinsed with 1× PBS + 0.05% Tween and biotinylated polyclonal goat anti-hNAP-2 IgG and anti-hPF-4 IgG detection antibodies (R&D Systems) were added at 0.2 µg/mL in 1× PBS for 2 h at room temperature with shaking. After three rinses in 1× PBS + 0.05% Tween, streptavidin-HRP (diluted 1:200 in 1% BSA in 1× PBS) was added and detected as described above.

### 4.8. Platelet Binding

EC monolayers were seeded on CellBIND surface 96-well plates and stimulated with 5 ng/mL TNF-α for four hours to create a proinflammatory environment. CellTracker Green CMFDA (5-chloromethylfluorescein diacetate) (Thermo Fisher Scientific) was dissolved in dimethyl sulfoxide (DMSO), diluted in EC media and added to ECs for 20 min at 37 °C and 5% CO_2_. Cells were then rinsed three times in TBS with 150 mM CaCl_2_ and 1% BSA in TBS with 150 mM CaCl_2_ was added to ECs for 10 min at room temperature to decrease nonspecific binding. ECs were then treated with 30 µM EC-SEAL variants for 1 h at 37 °C and 5% CO_2_. PRP was isolated from human whole blood as described above and labeled with CellTracker Orange CMRA (Thermo Fisher Scientific) (which was previously dissolved in DMSO) for 25 min at 37 °C and 5% CO_2_. Platelets were then pelleted via centrifugation for 10 min at 900× *g* and 25 °C, supernatant was removed and remaining platelets were suspended in EC media. Following EC-SEAL treatments and rinsing three times in TBS with 150 mM CaCl_2_, labeled platelets in EC media were added to ECs for 1 h at 37 °C and 5% CO_2_. After incubation, media was removed and the cells were rinsed in TBS with 150 mM CaCl_2_ and then fixed with 3% paraformaldehyde for 20 min at room temperature. Three final rinses with TBS with 150 mM CaCl_2_ were performed and cells were imaged using a Leica DMI6000 B microscope with EL6000 external light source (Leica Microsystems, Wetzler, Germany) and CoolSNAP HQ^2^ camera (Photometrics, Tucson, AZ, USA). Leica application suite (LAS) AF6000 software (Leica Microsystems) was utilized for image acquisition. Excitation/emission spectra used were 460–500 nm/512–542 nm for ECs (green) and 540–552 nm/580–620 nm for platelets (orange). Contrast and brightness editing of acquired images was accomplished using the program ImageJ (National Institutes of Health, Bethesda, MD, USA) and identical settings were applied to all images.

### 4.9. EC-SEAL Binding (Selectin Protein)

Recombinant human E-selectin (PeproTech) was dissolved in water and added to a 96-well EIA/RIA high binding plate at 0.1–100 µg/mL. Following overnight incubation at 4 °C, the plate was rinsed three times with TBS with 150 mM CaCl_2_ and blocked with 1% BSA in TBS with 150 mM CaCl_2_ for 1 h at room temperature with shaking. The plate was then rinsed three more times with TBS with 150 mM CaCl_2_. In experiments observing EC-SEAL binding, 3 µM EC-SEAL (in 1%BSA in TBS with 150 mM CaCl_2_) was added for 1 h at room temperature with shaking. Following three rinses with TBS with 150 mM CaCl_2_, streptavidin-HRP (1:200 in 1% BSA in TBS with 150 mM CaCl_2_) was added for 20 min at room temperature with shaking. For experiments observing antibody binding, primary mouse monoclonal anti-E-selectin IgG_2a_ antibody was added (1:50 dilution of initial 200 μg/mL in 1% BSA in TBS) for two hours at room temperature with shaking. Following three rinses with TBS with 150 mM CaCl_2_, secondary goat anti-mouse IgG HRP-conjugated antibody was added (1:1000 dilution of initial 0.5 mg/mL in 1% BSA in TBS) for 1 h at room temperature with shaking. HRP activity was detected as described above.

### 4.10. DVT Mouse Model

Ten-week-old C57BL/6 male mice (average weight: 26.4 g) were utilized in a model of surgically-induced deep vein thrombosis (DVT). All animals used in this study were obtained from Jackson Laboratory (Bar Harbor, ME) and fed standard chow diet. A well-established model of DVT caused by a significant inferior vena cava (IVC) flow restriction was utilized following a protocol (1505001247) approved by the institutional animal care and use committee at Purdue University. Mice were anesthetized using 1%–3% isoflurane and oxygen at a flow rate of 225 mL/min [58]; toe pinch was be used to determine sufficient anesthetic induction. Buprenorphine (0.03 mg/mL) was subcutaneously injected near the incision site at 0.05 mg/kg for pain management. Using aseptic technique, a small incision was made in the abdomen and the entrails were carefully exteriorized onto a sterile saline soaked gauze pad to prevent desiccation. The skin and organs were further retracted to fully expose the IVC, its branching vessels, and the infrarenal aorta. The aorta was then carefully separated from the IVC directly below the left renal vein. In an attempt to ligate all side and back branching vessels of the IVC, 6-0 black silk-braided sutures and a low power cautery pen (Bovie Medical, Purchase, NY, USA) were used, respectively. To achieve flow restriction, the proximal region of the IVC below the left renal vein was partially ligated by placing a 30-guage needle adjacent to the IVC and then tying a 6-0 black silk-braided suture around both the IVC and needle. The needle was then removed, resulting in an approximate 90% reduction in flow through the vessel. The muscle and skin layer were closed using 5-0 polypropylene sutures. A syringe with a 30-gauge needle was utilized to inject 100 µL of 30 μM EC-SEAL (*n* = 4), 200 IU/kg heparin (*n* = 6), or saline (*n* = 6) via tail vein. Tail vein injections were made approximately 10 min post-partial ligation of the IVC.

### 4.11. Ultrasound Imaging

Mice were anesthetized using 2%–3% isoflurane in 1.5 L/min oxygen. Cardiac and respiration rates were noninvasively monitored using gold-plated stage electrodes as part of the Vevo imaging station (FUJIFILM VisualSonics Inc., Toronto, ON, Canada). The mice were placed in a supine position on heated animal stage and the temperature was monitored using a rectal probe such that animals remained near 37°C. Depilatory cream was applied to remove hair on the abdomen prior to imaging, and warm ultrasound gel was applied on the skin to the entire abdomen just below the xiphoid process. IVC images were obtained using a Vevo2100 ultrasound imaging system with MS550D (40 MHz center frequency) and MS700 (50 MHz center frequency) transducers (FUJIFILM VisualSonics). The transducers were locked in the adjustable arm of the Vevo integrated rail system (FUJIFILM VisualSonics) for consistent image collection. In order to prevent distortion of the IVC, minimal pressure was applied to the abdomen to maintain original diameter of the vessel. The walls of the IVC were visualized with both long and short axes views. The angle of the stage was adjusted to optimize the view of the IVC and to minimize artifacts due to air and gas. Long and short axis B-mode images of the IVC were acquired at baseline (pre-operation) and at 6 h post-ligation of the IVC. Color Doppler images were used to assess blood flow through the IVC and branching vessels, and 3D scans of sequential slices were used to obtain vessel volumes as described previously [59,60] . Suture placement and the bifurcation of the IVC were used as landmarks to ensure consistent measurements between mice.

### 4.12. Ultrasound Image Analysis

All vessel measurements were made by the same ultrasonographer using Vevo LAB software (FUJIFILM VisualSonics). Thrombus length and volume and vessel volume were calculated using both the long axis and short axis ultrasound images. To determine the location of the thrombus, Color Doppler flow and B-mode images were utilized to visualize blood flow. The thrombus was also visualized as a hyperechoic region within the vessel lumen. Thrombus length was determined from the suture (point of IVC partial ligation) to the farthest point of thrombus and was calculated using an average of five measurements per image. The length of the thrombus in the 3D scans was compared to the length of thrombus in the long axis B-mode to ensure agreement. Thrombus volume was calculated by lofting together individual 2D segmentations from the first hyperechoic region in the lumen to the suture (where the lumen signal changed from hyperechoic to hypoechoic). Total IVC vessel volume was obtained using these same volumetric images and was measured from the suture to the IVC bifurcation. Percentage of IVC occlusion was obtained by determining the amount of thrombus volume within the total volume of the vessel.

### 4.13. Clotting Time (aPTT)

Activated partial thromboplastin time (aPTT) was measured using a Hemochron^®^ Response (Accriva Diagnostics, San Diego, CA, USA) with associated reagents. The protocol was run according to manufacturer’s instructions. Briefly, whole blood was obtained from healthy volunteers in sodium citrate collection vials and 2 mL was added to the Hemochron^®^ aPTT (citrated blood) test tube. One hundred microliters of saline, heparin or EC-SEAL was then added (final concentrations: 3.75–15 µg/mL heparin; 13–128 µg/mL EC-SEAL), and the test tube was shaken vigorously ten times from end-to-end. The test tube was then placed into the Hemochron^®^ Response device and clotting time was recorded at completion.

### 4.14. Statistics

Unless otherwise noted, all experiments were performed in triplicate or quadruplicate (*n* ≥ 2) and results are presented as mean ± standard deviation. Statistical analysis was performed using GraphPad Prism software (GraphPad Software, Inc., La Jolla, CA, USA). All results were analyzed using ANOVA with post-hoc Tukey test. Statistical significance threshold was set at *p* < 0.05.

## 5. Conclusions

Here, we present the development of a novel GAG derived, selectin targeting anti-adhesive coating (termed EC-SEAL) consisting of a dermatan sulfate backbone with multiple selectin-binding peptides designed to treat EC dysfunction. We demonstrated the ability of different EC-SEAL variants to successfully bind to selectin-expressing vascular ECs and SMCs. The most effective variants were examined further and DS-IEL_30_ was shown to preferentially bind to selectin protein and inhibit platelet binding and activation on inflamed EC surfaces. Additionally, DS-IEL_30_ reduced thrombus size in vivo in a DVT mouse model while exhibiting a minimal effect on clotting time. Thus, by binding to and pacifying the surface of inflamed endothelium, EC-SEAL has the potential to be utilized as a therapeutic in multiple diseases associated with endothelial dysfunction.

## Supplementary Materials

The following are available online at http://www.mdpi.com/1424-8247/10/2/36/s1, Figure S1: Selectin expression on ECs when stimulated with varying concentrations of TNF-α, Figure S2: Selectin expression on ECs when stimulated with varying concentrations of IL-1β, IL-6 and LPS, Figure S3: EC monolayer permeability in unstimulated conditions.

## References

[1] Brill A., Fuchs T.A., Chauhan A.K., Yang J.J., De Meyer S.F., Kollnberger M., Wakefield T.W., Lammle B., Massberg S., Wagner D.D. (2011). Von Willebrand factor-mediated platelet adhesion is critical for deep vein thrombosis in mouse models. Blood.

[2] Gawaz M., Langer H., May A.E. (2005). Platelets in inflammation and atherogenesis. J. Clin. Investig..

[3] Rajendran P., Rengarajan T., Thangavel J., Nishigaki Y., Sakthisekaran D., Sethi G., Nishigaki I. (2013). The vascular endothelium and human diseases. In. J. Biol. Sci..

[4] Sena C.M., Pereira A.M., Seica R. (2013). Endothelial dysfunction—A major mediator of diabetic vascular disease. Biochim. Biophys. Acta.

[5] O'Riordan E., Chen J., Brodsky S.V., Smirnova I., Li H., Goligorsky M.S. (2005). Endothelial cell dysfunction—The syndrome in making. Kidney Int..

[6] Davignon J., Ganz P. (2004). Role of endothelial dysfunction in atherosclerosis. Circulation.

[7] Dharmashankar K., Widlansky M.E. (2010). Vascular endothelial function and hypertension: Insights and directions. Curr. Hypertens. Rep..

[8] Briet M., Burns K.D. (2012). Chronic kidney disease and vascular remodelling: Molecular mechanisms and clinical implications. Clin. Sci..

[9] Franses J.W., Drosu N.C., Gibson W.J., Chitalia V.C., Edelman E.R. (2013). Dysfunctional endothelial cells directly stimulate cancer inflammation and metastasis. Int. J. Cancer. J. Int. Cancer.

[10] Hadi H.A.R., Carr C.S., Suwaidi J.A.I. (2005). Endothelial dysfunction—Cardiovascular risk factors, therapy, and outcome. Vasc. Health Risk Manag..

[11] Wu K.K., Thiagarajan P. (1996). Role of Endothelium in Thrombosis and Hemostasis. Annu. Rev. Med..

[12] Deanfield J.E., Halcox J.P., Rabelink T.J. (2007). Endothelial function and dysfunction: Testing and clinical relevance. Circulation.

[13] Reitsma S., Slaaf D.W., Vink H., van Zandvoort M.A., oude Egbrink M.G. (2007). The endothelial glycocalyx: Composition, functions, and visualization. Pflug. Archiv. Eur. J. Physiol..

[14] van den Berg B.M., Nieuwdorp M., Stroes E.S.G., Vink H. (2006). Glycocalyx and endothelial (dys) function—From mice to men. Pharmacol. Rep..

[15] Salmon A.H., Satchell S.C. (2012). Endothelial glycocalyx dysfunction in disease: Albuminuria and increased microvascular permeability. J. Pathol..

[16] Ait-Oufella H., Maury E., Lehoux S., Guidet B., Offenstadt G. (2010). The endothelium: Physiological functions and role in microcirculatory failure during severe sepsis. Intensive Care Med..

[17] Rumbaut R.E., Thiagarajan P. (2010). Chapter 2: General Characteristics of Platelets. Platelet-Vessel Wall Interactions in Hemostasis and Thrombosis.

[18] Gurney D., Lip G.Y.H., Blann A.D. (2002). A Reliable Plasma Marker of Platelet Activation—Does It Exist?. Am. J. Hematol..

[19] Massberg S., Enders G., Leiderer R., Eisenmenger S., Vestweber D., Krombach F., Messmer K. (1998). Platelet-Endothelial Cell Interactions During Ischemia-Reperfusion—The Role of P-Selectin. Blood.

[20] Smith C., Damas J.K., Otterdal K., Oie E., Sandberg W.J., Yndestad A., Waehre T., Scholz H., Endresen K., Olofsson P.S. (2006). Increased levels of neutrophil-activating peptide-2 in acute coronary syndromes: Possible role of platelet-mediated vascular inflammation. J. Am. Coll. Cardiol..

[21] Balakumar P., Koladiya R.U., Ramasamy S., Rathinavel A., Singh M. (2008). Pharmacological Interventions to Prevent Vascular Endothelial Dysfunction: Future Directions. J. Health Sci..

[22] Yu W., Akishita M., Xi H., Nagai K., Sudoh N., Hasegawa H., Kozaki K., Toba K. (2006). Angiotensin converting enzyme inhibitor attenuates oxidative stress-induced endothelial cell apoptosis via p38 MAP kinase inhibition. Clin. Chim. Acta Int. J. Clin. Chem..

[23] Jourde-Chiche N., Dou L., Cerini C., Dignat-George F., Brunet P. (2011). Vascular incompetence in dialysis patients--protein-bound uremic toxins and endothelial dysfunction. Semin. Dial..

[24] Fliser D. (2010). Perspectives in renal disease progression: The endothelium as a treatment target in chronic kidney disease. J. Nephrol..

[25] Ma S., Tian X.Y., Zhang Y., Mu C., Shen H., Bismuth J., Pownall H.J., Huang Y., Wong W.T. (2016). E-selectin-targeting delivery of microRNAs by microparticles ameliorates endothelial inflammation and atherosclerosis. Sci. Rep..

[26] Theoharis S., Krueger U., Tan P.H., Haskard D.O., Weber M., George A.J. (2009). Targeting gene delivery to activated vascular endothelium using anti E/P-Selectin antibody linked to PAMAM dendrimers. J. Immunol. Methods.

[27] Bachtarzi H., Stevenson M., Subr V., Ulbrich K., Seymour L.W., Fisher K.D. (2011). Targeting adenovirus gene delivery to activated tumour-associated vasculature via endothelial selectins. J. Control Release.

[28] Barthel S.R., Gavino J.D., Descheny L., Dimitroff C.J. (2007). Targeting selectins and selectin ligands in inflammation and cancer. Expert Opin. Ther. Targets.

[29] Haverslag R., Pasterkamp G., Hoefer I.E. (2008). Targeting Adhesion Molecules in Cardiovascular Disorders. Cardiovasc. Hematol. Disord. Drug Targets.

[30] Muzykantov V.R. (2013). Targeted Drug Delivery to Endothelial Adhesion Molecules. ISRN Vasc. Med..

[31] Fukuda M.N., Ohyama C., Lowitz K., Matsuo O., Pasqualini R., Ruoslahti E., Fukuda M. (2000). A Peptide Mimic of E-Selectin Ligand Inhibits Sialyl Lewis X-dependent Lung Colonization of Tumor Cells. Cancer Res..

[32] Fokong S., Fragoso A., Rix A., Curaj A., Wu Z., Lederle W., Iranzo O., Gatjens J., Kiessling F., Palmowski M. (2013). Ultrasound Molecular Imaging of E-Selectin in Tumor Vessels Using Poly *n*-Butyl Cyanoacrylate Microbubbles Covalently Coupled to a Short Targeting Peptide. Investig. Radiol..

[33] Leng X., Wang J., Carson A., Chen X., Fu H., Ottoboni S., Wagner W.R., Villanueva F.S. (2014). Ultrasound Detection of Myocardial Ischemic Memory Using an E-Selectin Targeting Peptide Amenable to Human Application. Mol. Imaging.

[34] Renkonen R., Fukuda M.N., Petrov L., Paavonen T., Renkonen J., Hayry P., Fukuda M. (2002). A Peptide Mimic of Selectin Ligands Abolishes In Vivo Inflammation But Has No Effect on the Rat Heart Allograft Survival. Transplantation.

[35] Raines E.W., Ferri N. (2005). Thematic review series: The immune system and atherogenesis. Cytokines affecting endothelial and smooth muscle cells in vascular disease. J. Lipid Res..

[36] Zeiffer U., Schober A., Lietz M., Liehn E.A., Erl W., Emans N., Yan Z.Q., Weber C. (2004). Neointimal smooth muscle cells display a proinflammatory phenotype resulting in increased leukocyte recruitment mediated by P-selectin and chemokines. Circ. Res..

[37] Yu G., Rux A.H., Ma P., Bdeir K., Sachais B.S. (2005). Endothelial expression of E-selectin is induced by the platelet-specific chemokine platelet factor 4 through LRP in an NF-kB-dependent manner. Blood.

[38] Cohen A.B., Stevens M.D., Miller E.J., Atkinson M.A.L., Mullenbach G. (1992). Generation of the neutrophil-activating peptide-2 by cathepsin G and Cathepsin G-treated human platelets. Am. J. Physiol. Lung Cell. Mol. Physiol..

[39] Schouten M., Wiersinga W.J., Levi M., van der Poll T. (2008). Inflammation, endothelium, and coagulation in sepsis. J. Leukoc. Biol..

[40] De Caterina R., Libby P., Peng H.B., Thannickal V.J., Rajavashisth T.B., Gimbrone J.M.A., Shin W.S., Liao J.K. (1995). Nitric Oxide Decreases Cytokine-induced Endothelial Activation. J. Clin. Investig..

[41] Zhang W.J., Stocker R., McCall M.R., Forte T.M., Frei B. (2002). Lack of inhibitory effect of HDL on TNFalpha-induced adhesion molecule expression in human aortic endothelial cells. Atherosclerosis.

[42] Tsou J.K., Gower R.M., Ting H.J., Schaff U.Y., Insana M.F., Passerini A.G., Simon S.I. (2008). Spatial regulation of inflammation by human aortic endothelial cells in a linear gradient of shear stress. Microcirculation.

[43] Pu F.R., Williams R.L., Markkula T.K., Hunt J.A. (2002). Expression of leukocyte-endothelial cell adhesion molecules on monocyte adhesion to human endothelial cells on plasma treated PET and PTFE in vitro. Biomaterials.

[44] van der Zijpp Y.J.T., Poot A.A., Feijen J. (2003). ICAM-1 and VCAM-1 expression by endothelial cells grown on fibronectin-coated TCPS and PS. J. Biomed. Mater. Res. A.

[45] Litwin M., Clark K., Noack L., Furze J., Berndt M., Albelda S., Vadas M., Gamble J. (1997). Novel Cytokine-independent Induction of Endothelial Adhesion Molecules Regulated by Platelet-Endothelial Cell Adhesion Molecule (CD31). J. Cell Biol..

[46] Luo J., Paranya G., Bischoff J. (1999). Noninflammatory Expression of E-Selectin Is Regulated by Cell Growth. Blood.

[47] Martens C.L., Cwirla S.E., Lee R.Y.W., Whitehorn E., Chen E.Y.F., Bakker A., Martin E.L., Wagstrom C., Gopalan P., Smith C.W. (1995). Peptides Which Bind to E-selectin and Block Neutrophil Adhesion. J. Biol. Chem..

[48] Goh C.S., Milburn D., Gerstein M. (2004). Conformational changes associated with protein-protein interactions. Curr. Opin. Struct. Biol..

[49] Keskin O. (2007). Binding induced conformational changes of proteins correlate with their intrinsic fluctuations: A case study of antibodies. BMC Struct. Biol..

[50] Cooke R.M., Hale R.S., Lister S.G., Shah G., Weir M.P. (1994). The Conformation of the Sialyl Lewis X Ligand Changes upon Binding to E-Selectin. Biochemistry.

[51] Preston R.C., Jakob R.P., Binder F.P., Sager C.P., Ernst B., Maier T. (2016). E-selectin ligand complexes adopt an extended high-affinity conformation. J. Mol. Cell Biol..

[52] Diaz J.A., Obi A.T., Myers D.D., Wrobleski S.K., Henke P.K., Mackman N., Wakefield T.W. (2012). Critical review of mouse models of venous thrombosis. Arterioscler. Thromb. Vasc. Biol..

[53] Myers D.D., Rectenwald J.E., Bedard P.W., Kaila N., Shaw G.D., Schaub R.G., Farris D.M., Hawley A.E., Wrobleski S.K., Henke P.K., Wakefield T.W. (2005). Decreased venous thrombosis with an oral inhibitor of P selectin. J. Vasc. Surg..

[54] Dhahri M., Mansour M.B., Bertholon I., Ollivier V., Boughattas N.A., Hassine M., Jandrot-Perrus M., Chaubet F., Maaroufi R.M. (2010). Anticoagulant activity of a dermatan sulfate from the skin of the shark Scyliorhinus canicula. Blood Coagul. Fibrinolysis.

[55] Duan J., Kasper D.L. (2011). Oxidative depolymerization of polysaccharides by reactive oxygen/nitrogen species. Glycobiology.

[56] van Golen R.F., Reiniers M.J., Vrisekoop N., Zuurbier C.J., Olthof P.B., van Rheenen J., van Gulik T.M., Parsons B.J., Heger M. (2014). The mechanisms and physiological relevance of glycocalyx degradation in hepatic ischemia/reperfusion injury. Antioxid. Redox Signal..

[57] Zhang J., Nakayama J., Ohyama C., Suzuki M., Suzuki A., Fukuda M., Fukuda M.N. (2002). Sialyl Lewis X-dependent Lung Colonization of B16 Melanoma Cells through a Selectin-like Endothelial Receptor Distinct from E- or P-Selectin. Cancer Res..

[58] Damen F.W., Adelsperger A.R., Wilson K.E., Goergen C.J. (2015). Comparison of Traditional and Integrated Digital Anesthetic Vaporizers. J. Am. Assoc. Lab. Anim. Sci..

[59] Phillips E.H., Yrineo A.A., Schroeder H.D., Wilson K.E., Cheng J.X., Goergen C.J. (2015). Morphological and Biomechanical Differences in the Elastase and AngII apoE^−/−^ Rodent Models of Abdominal Aortic Aneurysms. Biomed. Res. Int..

[60] Phillips E.H., Di Achille P., Bersi M.R., Humphrey J.D., Goergen C.J. (2017). Multi-Modality Imaging Enables Detailed Hemodynamic Simulations in Dissecting Aneurysms in Mice. IEEE Trans Med Imaging.

