# Supplementary material for: Development of a Glycosaminoglycan Derived, Selectin Targeting Anti-Adhesive Coating to Treat Endothelial Cell Dysfunction
Source: Pharmaceuticals (Basel). 2017 Mar 29;10(2):36. doi: 10.3390/ph10020036 (PMC5490393; doi:10.3390/ph10020036)
Supplement: Supplementary file 1 [file pharmaceuticals-10-00036-s001.pdf]

pharmaceuticals

Supplemental Materials: Development of a Glycosaminoglycan Derived, Selectin Targeting Anti-Adhesive Coating to Treat Endothelial Cell Dysfunction

James R. Wodicka [1,2], Andrea M. Chambers [1], Gurneet S. Sangha [1], Craig J. Goergen [1] and Alyssa Panitch [1,3,*]

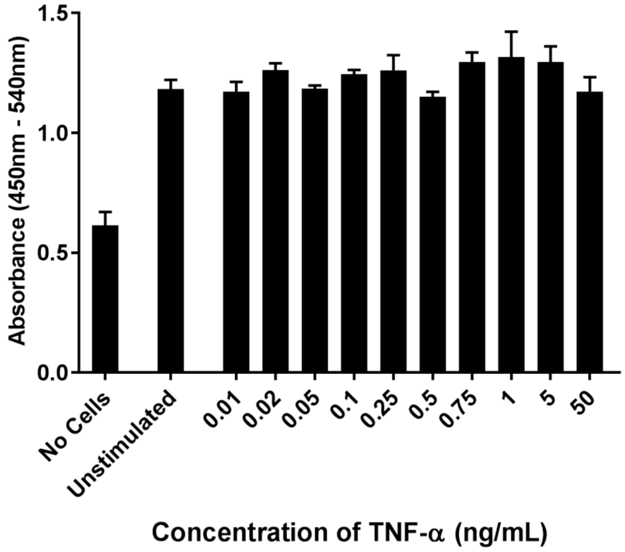

Figure S1. Selectin expression on ECs when stimulated with varying concentrations of TNF-α. Expression was quantified using primary anti-E-selectin and secondary HRP-conjugated antibodies. Selectin levels did not change when stimulated with TNF-α. $n = 3$; $p < 0.05$.

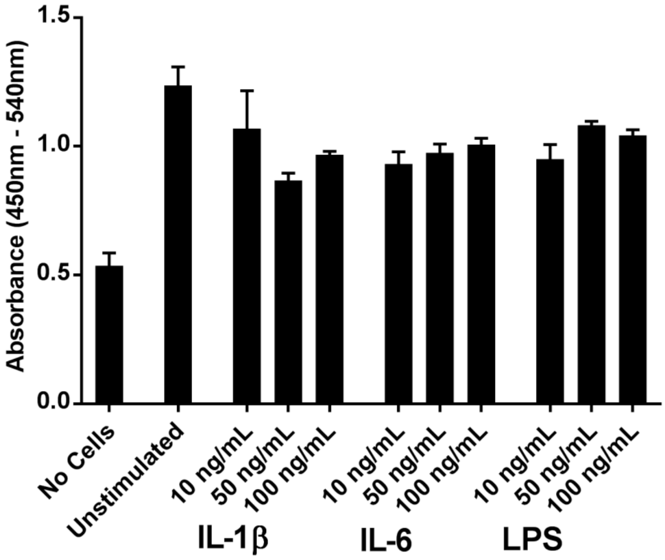

Figure S2. Selectin expression on ECs when stimulated with varying concentrations of IL-1β, IL-6 and LPS. Expression was quantified using primary anti-E-selectin and secondary HRP-conjugated antibodies. Proinflammatory stimuli failed to increase selectin expression on the EC surface. $n = 3$; $p < 0.05$.

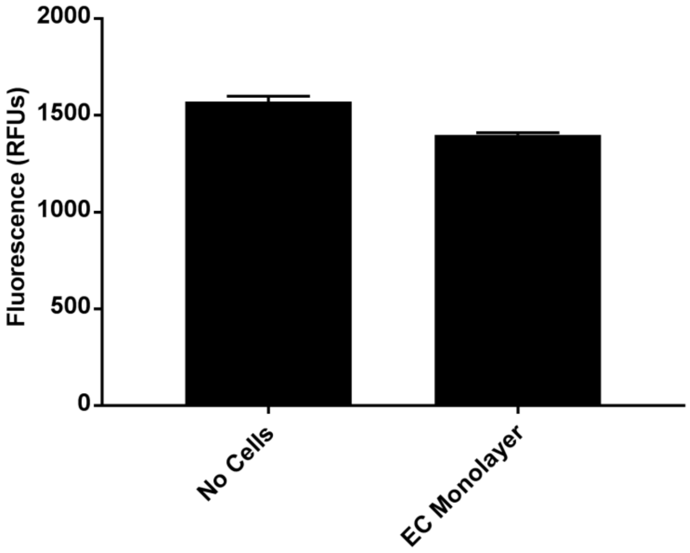

Figure S3. EC monolayer permeability in unstimulated conditions. RITC-dextran was added to the upper chamber of Transwells with and without an EC monolayer. Following incubation, a sample from the lower chamber was collected and fluorescence (Ex: 520 nm; Em: 590 nm) was recorded. Despite ECs present, a majority of the RITC-dextran was able to pass through the Transwell, indicating that the monolayer was not fully intact. $n = 2$; $p < 0.05$.
